# SAS-SEINet: A SNR-Aware Adaptive Scalable SEI Neural Network Accelerator Using Algorithm–Hardware Co-Design for High-Accuracy and Power-Efficient UAV Surveillance †

**DOI:** 10.3390/s22176532

**Published:** 2022-08-30

**Authors:** Jiayan Gan, Ang Hu, Ziyi Kang, Zhipeng Qu, Zhanxiang Yang, Rui Yang, Yibing Wang, Huaizong Shao, Jun Zhou

**Affiliations:** 1 School of Information and Communication Engineering, University of Electronic Science and Technology of China, Chengdu 611731, China; 2 Research Center of Advanced RF Chips and Systems, Nanhu Laboratory, Jiaxing 314000, China

**Keywords:** UAV, SEI, DCNN, SNR, power efficiency

## Abstract

As a potential air control measure, RF-based surveillance is one of the most commonly used unmanned aerial vehicles (UAV) surveillance methods that exploits specific emitter identification (SEI) technology to identify captured RF signal from ground controllers to UAVs. Recently many SEI algorithms based on deep convolution neural network (DCNN) have emerged. However, there is a lack of the implementation of specific hardware. This paper proposes a high-accuracy and power-efficient hardware accelerator using an algorithm–hardware co-design for UAV surveillance. For the algorithm, we propose a scalable SEI neural network with SNR-aware adaptive precision computation. With SNR awareness and precision reconfiguration, it can adaptively switch between DCNN and binary DCNN to cope with low SNR and high SNR tasks, respectively. In addition, a short-time Fourier transform (STFT) reusing DCNN method is proposed to pre-extract feature of UAV signal. For hardware, we designed a SNR sensing engine, denoising engine, and specialized DCNN engine with hybrid-precision convolution and memory access, aiming at SEI acceleration. Finally, we validate the effectiveness of our design on a FPGA, using a public UAV dataset. Compared with a state-of-the-art algorithm, our method can achieve the highest accuracy of 99.3% and an F1 score of 99.3%. Compared with other hardware designs, our accelerator can achieve the highest power efficiency of 40.12 Gops/W and 96.52 Gops/W with INT16 precision and binary precision.

## 1. Introduction

With the rapid development of 5G and beyond (e.g., 6G) and wireless communication technology and the increasing complexity of the electromagnetic environment, unmanned aerial vehicles (UAVs), also known as drones, have received increasing popularity, since they offer extraordinary ability, high mobility, and low cost in aiding and improving a wireless network. For instance, an UAV can provide flexible and stable connectivity between communication devices, establish relay links [1] and cellular networks [2], assist radio localization and navigation [3], and so on [4,5,6]. While benefiting from an UAV, the misuse and “black flight” of drones is also a concern. Some civilian drones may enter restricted areas without certification, which will seriously threaten airspace security, cybersecurity, and even public safety. Despite the government’s drone regulation efforts, such as real-name registration and electronic fences, there are still some illegal drones that violate these regulatory measures.

To address the above security issue, UAV surveillance systems have been developed to detect and identify different UAVs. As one of the surveillance methods, the RF-based specific emitter identification (SEI) technology [7,8] can identify a captured RF signal from UAVs to the controllers and distinguish different UAV individuals by extracting the electromagnetic signal characteristics, known as a RF fingerprint. The RF fingerprint [9,10] is an inherent feature caused by the imperfections of a RF circuit including I/Q imbalance, phase noise, frequency offset, etc. These imperfections have unique and inevitable characteristics, which are hard to imitate. At the same time, the RF fingerprint contains rich information about UAVs, which allows RF-based surveillance to be less-constrained in many scenarios. To obtain such a RF fingerprint, SEI based on machine learning (ML) [11,12], especially a deep convolutional neural network (DCNN) [13,14,15,16,17], can automatically extract deeper features of RF signals with high accuracy, which has attracted many researchers. However, these methods have high computational complexity, requiring a domain-specific DCNN processor for acceleration. Although some DCNN processors have been proposed, most of them are used in computer vision and other fields, which are not suitable for SEI in terms of power efficiency.

In this paper, we propose a high-accuracy and power-efficient SEI accelerator using an algorithm–hardware co-design for UAV surveillance [18]. The main contributions are described as follows:

- On the algorithm level, scalable SEI neural network with SNR-aware adaptive precision computation is proposed to deal with the UAV identification task under different SNRs. Then, a 16-bit DCNN and a binary DCNN are used for low SNR and high SNR, respectively. Two DCNNs can be adaptively switched according to the SNR estimated by the second and fourth moments (M2M4) algorithm, which can reduce the power consumption while ensuring the accuracy.
- On the algorithm level, a short-time Fourier transform (STFT)-based feature extraction, reusing the DCNN method, is proposed to pre-extract features of a UAV signal. It allows the reuse of the convolution operators of a DCNN and reduces hardware costs. In addition, we use normalization, quantization, and denoising preprocessing methods to improve the overall accuracy.
- On the hardware level, a DCNN engine with hybrid-precision convolution and memory access is proposed, which speeds up the computation and reduces hardware costs. The hybrid-precision convolution can be reused by the convolution, binary convolution, and STFT convolution operation. The hybrid-precision memory access can reuse the parameter storge of a DCNN and a binary DCNN.
- On the hardware level, the specialized SNR sensing engine and denoising engine are designed for SEI. Denoising engine is responsible for denoising the RF data to reduce signal redundancy. A SNR sensing engine is responsible for estimating the SNR of the emitter signal, which determines whether we use a DCNN or abinary DCNN.
- The rest of this paper is organized as follows: Section 2 reviews the related work and techniques; Section 3 focuses on the proposed algorithm–hardware co-design for a SEI accelerator; Section 4 describes the dataset, neural network architecture, evaluation method, test setup, and experimental results; Section 5 compares our method with other algorithms and hardware designs; and Section 6 presents the conclusions.

## 2. Related Works

Recently, many ML-based SEI methods have been proposed for UAV surveillance. Al-Sa’d et al. [14] first built a public RF-based UAV dataset, including the RF data of different UAVs in different flight modes, such as: off mode, on and connected mode, hovering mode, and flight and video recording mode. To confirm the feasibility of the above RF-based UAV dataset, they designed three four-layer deep neural networks (DNN), with the same architecture for detecting the presence of UAVs, the presence of UAVs and their types, and, finally, the presence of UAVs, their types, and their flight modes. The classification categories for the three tasks are 2, 4, and 10 categories, respectively. As the difficulty of the classification task gradually increases, the accuracy of the first DNN (class 2) decreases from 99.7% to 84.5% in the next DNN (class 4) and, finally, to 46.8% in the third DNN (class 10).

Al-Emadi et al. [15] used a six-layer DCNN for UAV detection and an eight-layer DCNN for UAV-type classification and flight-pattern classification. A one-dimensional convolution layer, one-dimensional pooling layer, and dense layer form their DCNN. Compared with a DNN, a DCNN can achieve better accuracy of 99.8%, 85.8%, and 59.2%, respectively.

Allahham et al. [16] proposed a data channelization preprocessing method and multi-channel one-dimensional DCNN architecture. For preprocessing, they divided the full Wi-Fi frequency spectrum (80 MHz for the 13 overlapping channels) into 8 equal-bandwidth channels via data channelization technology. For multi-channel one-dimensional DCNN, the network is a five-layer structure, with multichannel inputs that correspond to each separated spectrum channel. After the training process, their method can learn and analyze the feature of the different frequency band RF data, which improved the accuracy of three classification task by 0.2%, 8.8%, and 28.2%, respectively.

In [17], a multi-channel deep neural network with a joint feature engineering generator method is proposed by Yang et al. Unlike the multi-channel DCNN in the previous paper, their two-channel neural network extracts the features of the high-frequency and low-frequency components separately in the shallow layer and fuses them before the final, fully connected layer. For the feature-engineering generator, data truncation and the moving average filter are utilized to remove the noise effects. Separated normalization is utilized to train the neural network more easily rather than normalizing together, which prevents the smaller-valued high-frequency component from being dominated by the low-frequency component. Experiments shows the effectiveness of their method, which improves the accuracy of 10 categories to 98.2%.

Nemer [12] et al. propose a hierarchical learning approach for UAV identification and detection. Specifically, three tasks use three levels of ensemble learning classifiers in a cascaded form, which include a classifier for detecting UAVs in the first level, a classifier for detecting UAV types in the second level, and the last two classifiers for detecting Bebop and AR UAV flight patterns in the third level. The KNN and XGBoost classifiers form an ensemble classifier, with a final output that is based on the voting of the outputs from these two classifiers. The results show that their method can detect the presence of an UAV and identify the type of an UAV and the corresponding flight pattern with an average accuracy of about 99.2%.

Along with the extensive research to improve the accuracy of UAV identification algorithms, hardware implementation is also a key part of SEI-based UAV surveillance deployment. Similar to our work, Soltani et al. [19,20] designed an embedded implementation of a deep-learning-based classifier named DeepRadio for the modulation classification of RF signals. Unlike SEI, this classifier classifies the received signals into different modulation types. In their experiments, the DeepRadio successfully identifies the different modulation types of one USRP N210 with high accuracy and low power consumption.

In general, the existing algorithms focus only on the improvement of accuracy and lack algorithm–hardware co-design, which is not hardware-friendly. If such algorithm is directly applied to the hardware, it may bring high power consumption. Although some general-purpose processors (e.g., CPUs and GPUs) and ML-based processors are available, they are not specifically optimized for SEI and, therefore, cannot meet the real-time or power-efficiency requirements of state-of-the-art algorithms.

## 3. Proposed Algorithm–Hardware Co-Design for SEI Accelerator

**Figure 1:**
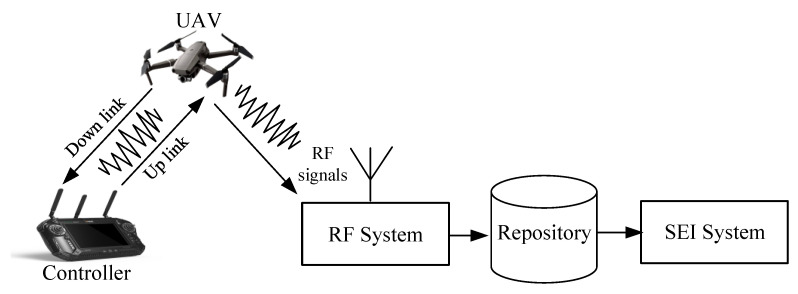
Block diagram of UAV surveillance platform.

The SEI-based UAV surveillance platform is shown in Figure 1. It consists of an UAV, a remote controller, a RF system, a repository, and a SEI system. RF systems such as universal software radio peripherals (USRP) can collect unique RF signals from different types of UAVs with different flight modes by passively and continuously listening to the communication between the UAV and the remote controller, which includes control command signals (controller to UAV), telemetry signals, and video signals (UAV to controller). The captured RF signals are then stored in a local database repository, and the stored data can be analyzed by a SEI system to detect the presence of the drone, the type of drone, and the flight pattern of the drone.

### 3.1. SNR-Aware Adaptive Scalable SEI Neural Network

**Figure 2:**
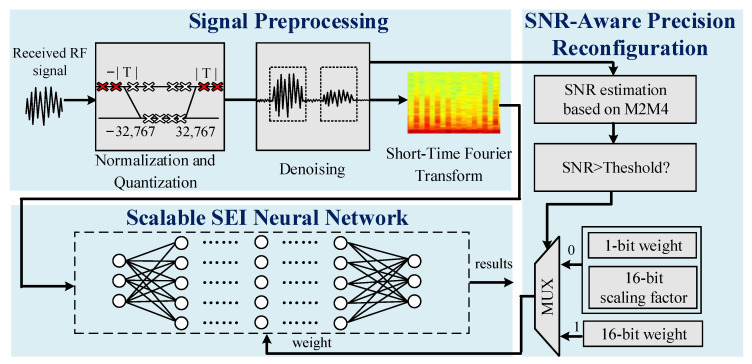
SNR-Aware adaptive Scalable SEI neural Network.

For the SEI algorithm, the most conventional method based on deep learning is to use a uniform high-precision type of neural network for the classification, such as INT16, FLOAT32, and even FLOAT64 [21]. If this kind of SEI algorithm is directly applied to hardware, it will bring more power consumption in simple occasions with low precision requirements. In other deep-learning-application fields, binary DCNN has shown great advantages in low precision, which can reduce the overall hardware overhead. Here, aiming at the application of SEI, we take the advantages of binary DCNN and propose our SNR-Aware adaptive Scalable SEI neural Network (SAS-SEINet), as shown in Figure 2.

The SAS-SEINet algorithm includes the signal preprocessing, SNR-aware precision reconfiguration, and scalable SEI neural network. Signal preprocessing is responsible for normalization, quantization, denoising, and STFT. SNR-aware precision reconfiguration is performed to estimate the SNR based on the second and fourth moments (M2M4) algorithm and adjust the neural network precision according to the threshold judgment. The scalable SEI neural network, also denoted as SEI-DCNN, identifies the different emitter signals ranging with different SNRs. For low SNR, the SEI neural network is the conventional DCNN with INT16 precision, which can maintain the accuracy. For high SNR, the binary DCNN is applied to reduce power consumption, while the accuracy does not drop too much.

#### 3.1.1. Scalable SEI-DCNN with SNR-Aware Adaptive Precision Computation

**Figure 3:**
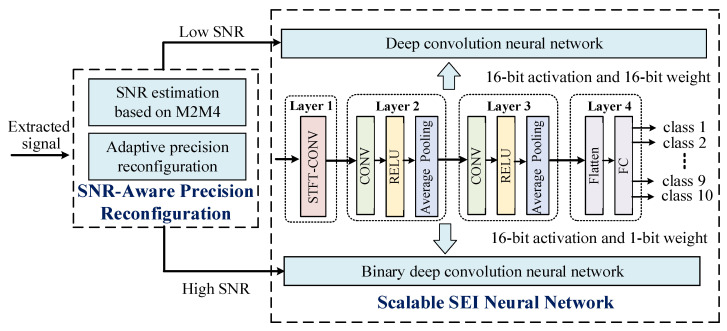
Scalable SEI-DCNN with SNR-aware adaptive precision computation.

Conventional DCNN with fixed high-precision parameters brings high memory usage and power consumption. Those “simple” samples can be classified well with lower precision parameters. Especially for RF signals, signals with high SNR are easier to recognize than those with low SNR. Therefore, the precision with adaptive reconfiguration is more suitable for processing signals with different SNRs. Based on the above, we propose a scalable SEI-DCNN with SNR-aware adaptive precision computation, as shown in Figure 3. Under the control of SNR-aware precision reconfiguration, the precision of SEI neural networks can be reconfigured toward different SNR. To maintain the accuracy at low SNR, the backbone neural network is a conventional DCNN with 16-bit activation and 16-bit weight. To reduce the algorithm complexity and power consumption at high SNR, the backbone neural network is a binary DCNN with 16-bit activation and 1-bit weight.

SNR-aware precision reconfiguration consists of SNR estimation based on M2M4 and adaptive precision reconfiguration. The M2M4 method proposed in [22] successfully estimates the carrier strength and noise strength in a complex AWGN channel. Since the M2M4 does not need carrier recovery and has a wide range of SNR estimation, it is more suitable for practical applications. After the estimation of M2M4, the estimated SNR is sent to the adaptive precision reconfiguration, based on a simple judgment mechanism. If the estimated SNR is greater than a threshold, the binary DCNN will be used to process such signals with high SNR. Otherwise, the DCNN will be used to process signal with low SNR. The threshold in judgment mechanism is obtained from the experiment, which can be referred to in Section 4.5.

Specifically, the expression of SNR estimation based on M2M4 is as follows:

(1) $$SNR=\frac{Es}{N}=\frac{\sqrt{2M_{2}^{2}-M_{4}}}{M_{2}-\sqrt{2M_{2}^{2}-M_{4}}}={\left(\sqrt{\frac{1}{2-\left(\frac{M_{4}}{M_{2}^{2}}\right)}}-1\right)}^{-1}$$

where *M*_2_ and *M*_4_ represent the second and the fourth moments of the received signal $y_{n}$. Due to the fact that the statistical average of received signals cannot be obtained directly, one general method is to approximate the statistical mean by time average, as follows:

(2) $$\{\begin{array}{l} M_{2}\approx \frac{1}{N}\sum_{n=0}^{N-1}{\left|y_{n}\right|}^{2}\\ M_{4}\approx \frac{1}{N}\sum_{n=0}^{N-1}{\left|y_{n}\right|}^{4} \end{array}$$

With the increase in the number of observation data (denoted as *N*), the SNR value estimated by M2M4 is closer to the real value. In addition, it is found that the standard deviation of SNR estimation is less than 0.2 dB when the *N* is more than 2000.

For scalable SEI-DCNN, its backbone neural network is a four-layer convolutional neural network, including a STFT convolution layer, two convolutional layers, and a fully connected (FC) layer. Each of convolutional layers consists of convolution, activation function, and average pooling.

- Convolution layer

In a conventional DCNN [23], the common operation of a convolution layer can be expressed as follows:

(3) $$A_{n+1}=p\left(f\left(A_{n}\;⊗\;W_{n}\right)\right)$$

where $A_{n+1}$ denotes the (*n* + 1)th layer output tensor generated by the previous layer tensor $A_{n}$ and corresponding weight tensor $W_{n}$. f denotes the activation function (e.g., sigmoid and ReLu), which introduces the non-linearity to the model. p denotes the pooling function, which compresses the activation values and removes the redundant information. Here, we use the ReLu activation and average pooling function. Standard convolution operation ⊗ includes multiplication and addition operations, which occupies the majority computation of the DCNN.

**Figure 4:**
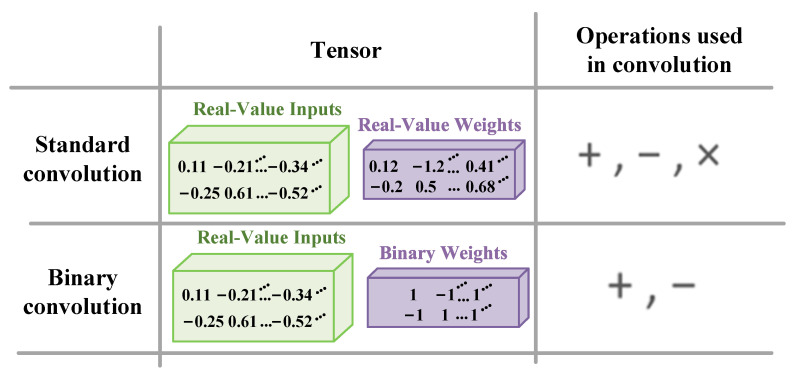
The comparison between standard convolution and binary convolution.

Unlike DCNN, the standard convolution is replaced by binary convolution in a binary DCNN [24], as shown in Figure 4. Binary convolution uses binary weights for the convolution, which is implemented by addition and subtraction operations instead of multiplication operations. Thus, a binary DCNN greatly reduces the memory usage and computation. Specifically, the convolution operation in a binary DCNN can be transformed as follows:

(4) $$A_{n}\;⊗\;W_{n}\approx \left(A_{n}⊕BW_{n}\right)\alpha$$

where ⊕ represents binary convolution without any multiplication. The binarized weight $BW_{n}$ is derived from $W_{n}$. The scale factor α introduces a small amount of multiplication, but it will improve the overall classification accuracy. For the convolution layer, different scale factors are used to multiply the convolution results of each output channel. For the fully connected layer, the scale factor of the output neuron itself is used to multiply the result of matrix multiplication.

The optimal value of the binary weight $BW_{n}$ used in the binary neural network can be obtained by taking the sign of the original weight $W_{n}$.

(5) $$BW_{n}=sign\left(W_{n}\right)$$

The optimal value of the scale factor α is obtained by averaging the sum of the absolute values of the elements $w_{n}^{i}$ in the original weight tensor $W_{n}$ is expressed as:

(6) $$\alpha =\frac{\sum^{\;}\left|w_{n}^{i}\right|}{n}=\frac{1}{n}{∥w_{n}∥}_{l1}$$

- FC layer

The FC layer [23] acts as the classifier and is usually located in the last layers of the DCNN. Unlike the previous layer that maps the initial input to the hidden space to extract features, the FC layer maps the learned hidden features to the label space. Specifically, the operation of FC layer can be expressed as:

(7) $$A_{n+1}=f\left(A_{n}\times W_{n}\right)$$

where × denotes the matrix multiplication operation between previous layer tensor $A_{n}$ and corresponding weight tensor $W_{n}$. f denotes the activation function (e.g., sigmoid and softmax), which produces the score or probability of each category. Here, the activation function is available for DCNN training. During inference phase, the matrix multiplication can be replaced by a full convolution operation, and the activation function can be optionally skipped, which does not affect the final classification result.

- STFT convolution layer

As one of the widely used preprocessing method, STFT can map a one-dimensional time-domain signal into a joint distribution of time and frequency, preserving both the time-domain and frequency-domain features of the signal. In this paper, we merged the STFT preprocessing into the DCNN as a convolution layer. The details of this method will be introduced in the following section.

#### 3.1.2. STFT-Based Feature Extraction Reusing DCNN

Although a DCNN has powerful automatic feature-extraction capabilities, directly feeding raw data without any processing into neural network may make training difficult to converge and result in poor performance. Therefore, researchers often perform appropriate preprocessing on RF signals to improve the overall accuracy of the algorithm. In this paper, we propose a STFT reusing a DCNN method to extract the feature. STFT is implemented as a STFT convolution layer, which allows the reuse of the DCNN and reduces hardware costs.

Given a window function ω with length *N* and stride *s*, the standard STFT [25] amplitude spectrum $\left|X_{stft}\left(t,f\right)\right|$ of original signal x can be expressed as:

(8) $$\left|X_{stft}\left(t,f\right)\right|=\left|\overset{\infty }{\underset{n=-\infty }{\sum }}x\left[n\right]\omega \left[n-st\right]e^{-i\frac{2\pi n}{N}f}\right|$$

To make the computation of STFT more convenient, we derive Equation (8) as:

(9) $$\left|X_{stft}\left(t,f\right)\right|=\left|(x\left[t\right]⊗{\overset{STFT\;kernel}{\overbrace{\omega \left[t\right]e^{-i\frac{2\pi t}{N}f})}}}_{with\;stride\;s}\right|\;$$

where ⊗ denotes the convolution operation between x and the STFT kernel. With the Euler formula, the complex STFT kernel can be split into real and imaginary parts:

(10) $$\begin{array}{c} K_{real}\left(t,f\right)=\omega \left[t\right]\cos\left(2\pi tf/N\right)\\ K_{imag}\left(t,f\right)=-\omega \left[t\right]\sin\left(2\pi tf/N\right) \end{array}$$

By substituting Equation (10) into Equation (9), we can obtain:

(11) $$\left|X_{stft}\left(t,f\right)\right|=\left|x\left[t\right]⊗K_{real}\left(t,f\right)+i\times x\left[t\right]⊗K_{imag}\left(t,f\right)\right|$$

where ⊗ denotes the convolution operation. It can be seen that the formulation of the STFT amplitude spectrum can be expressed as the combination of two one-dimensional convolutions. In other words, we can use two one-dimensional convolutions with the $K_{real}$ and $K_{imag}$ kernels to compute the STFT amplitude spectrum instead, which reuses our convolution operator of DCNN and is easier to implement.

In addition to STFT, normalization, quantization, and denoising preprocessing methods are also used to process raw data. These methods are described in detail as follows:

- Normalization and quantization

Normalization [26] is the process of scaling individual samples to have a unit norm. It makes all samples have the same range and facilitates convergence of training. In this paper, we use min–max normalization to scale the RF samples to the range of [−1, 1]. Specifically, our normalization is formulated as follows:

(12) $$X_{min-max}=\frac{2\left(x-x_{min}\right)}{x_{max}-x_{min}}-1$$

where *x* and $X_{min-max}$ are the input RF data and the output quantized data, respectively. The minimum and maximum values of x are denoted by $x_{min}$ and $x_{max}$.

For quantization, we utilized the INT16 quantization based on Kullback–Leibler divergence (KLD) to quantize the input data. Such a method [27] attempts to approximate the original numerical distribution of FP32 with that of INT16, which ensures the accuracy of the network after quantization and facilitates deployment of FPGA. Specifically, the quantization is formulated as follows:

(13) $$Z_{quantization}=\frac{(2^{15}-1)\times clip\left(z,-\left|T\right|,\left|T\right|\right)}{\left|T\right|}.\;$$

where *z* and $Z_{quantization}$ denote the original FP32 data and the quantized INT16 data, respectively. |T| is the saturation threshold of quantization, and clip(z,−|T|,|T|) is the function that truncates the original data z to the range of [−|T|,|T|]. Generally, the threshold |T| is less than the maximum of $\left|z_{min}\right|$ and $\left|z_{\max}\right|$.

Instead of directly mapping the range of [$z_{min}$, $z_{max}$] to [−$\left(2^{15}-1\right)$, $\left(2^{15}-1\right)$], KLD-based quantization truncates the values outside the ±|T| and maps the range of [−|T|, |T|] to [−$\left(2^{15}-1\right)$, $\left(2^{15}-1\right)$], which prevents the accuracy from being affected by the abnormal $z_{max}$ and $z_{min}$. Additionally, the quantization tries to adjust the threshold |T| to approximate the distribution of the INT16 data to the original distribution of the FP32 data. The distribution similarity can be measured by the KLD. The smaller the KLD value, the more similar the two distributions are, and the best threshold |T| can be obtained when the KLD value is minimal.

- Denoising

**Figure 5:**
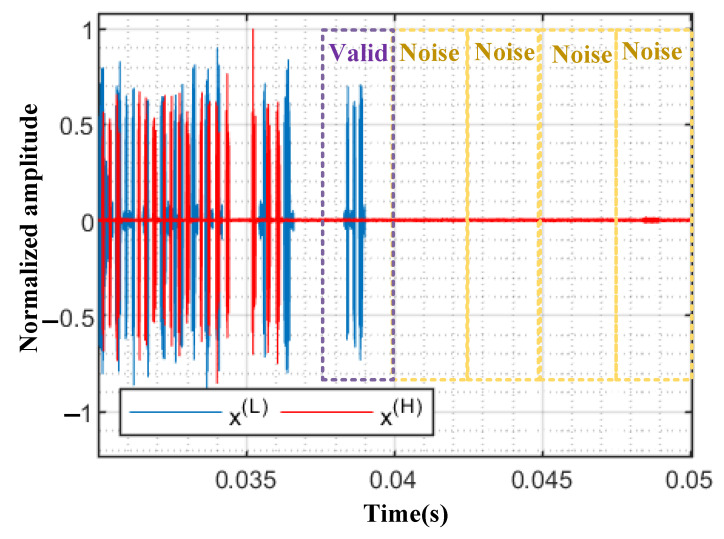
The noise in raw RF data.

Raw RF data often include long segments of noise, as shown in Figure 5, and such segments may dominate the entire signal, bringing confusion to the training of model. To reduce the effect of background noise, denoising is an effective means to separate the UAV signal from the noise. Since the amplitude of noise is much smaller than that of the signal, we utilize a short-time energy detection to extract the signal [28]. Specifically, the short-time energy $E_{m}$ can be calculated as follows:

(14) $$E_{m}=\overset{\infty }{\underset{n=-\infty }{\sum }}{\left(x\left[n\right]\omega \left[m-n\right]\right)}^{2}$$

where *x* and ω are the input data and window function, respectively. When the short-time energy $E_{m}$ in the window is higher than a certain threshold, we can judge that there is a valid signal in the window, otherwise there is noise.

### 3.2. Reconfigurable Hybrid-Precision SEI Hardware Accelerator

**Figure 6:**
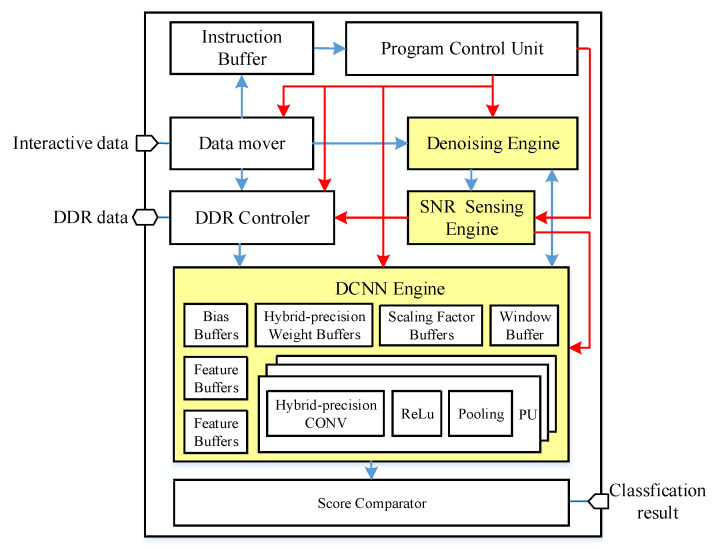
The architecture of proposed SEI accelerator.

The architecture of the proposed SEI hardware accelerator is shown in Figure 6. The program control unit (PCU) reads the user instructions (e.g., SNR threshold, DCNN structure) from the instruction buffer and controls the SEI acceleration to achieve programmability. The data mover and DDR controller are responsible for the data transmission between on-chip and off-chip. Once the storage capacity of the on-chip buffer exceeds, the off-chip DDR can be used for storage. The score comparator obtains the results of the last layer of output neurons and produces the final classification result.

Dedicated to the SEI algorithm, we design three core computation modules including a denoising engine, SNR sensing engine, and DCNN engine. The denoising engine interacts with the window buffers that store the window function data and is responsible for denoising the RF data to reduce signal redundancy. The SNR sensing engine is responsible for estimating the SNR of the emitter signal, which determines whether we use a DCNN or a binary DCNN. For DCNN processing, the double feature buffers, hybrid-precision weight buffers, bias buffers, and scaling factor buffers are used to store the input–output feature map data and trained parameters of DCNN. Under the condition of sufficient storage capacity, these buffers can store a limited-size DCNN fully on-chip without having to go off-chip, which reduces the latency and power consumption. In particular, the hybrid-precision weight buffers can store both 16-bit weight or binary weight in a compact-storage strategy, and, thus, a larger neural network can be stored in binary weight mode. Besides, the DCNN engine includes multiple processing units (PU). Each PU is composed of a hybrid-precision CONV, ReLu, and pooling module. Instead of designing two computation units separately, the hybrid-precision CONV can compute a 16-bit convolution or binary convolution in a multiplexed manner. In addition, the DCNN engine can process multiple output feature maps or multiple input feature maps with two parallel modes, respectively, according to the different characteristics of convolution and full connection (FC) computing.

#### 3.2.1. DCNN Engine with Hybrid-Precision Convolution and Memory Access

**Figure 7:**
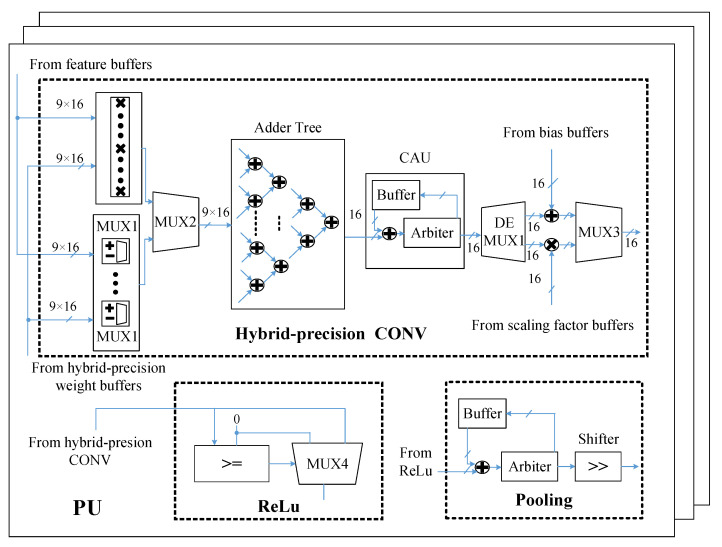
The core processing module of DCNN engine.

The core processing module of DCNN engine is shown in Figure 7, including multiple PUs with a hybrid-precision CONV, ReLu, and pooling modules. For a hybrid-precision CONV module, both standard convolutional computation and binary convolutional computation can be supported. Taking 3 × 3 convolution as an example, in the standard convolution mode, nine groups of multipliers will be selected by MUX2 to calculate nine times of multiplication with the feature maps and weights in the convolution. In the binary convolution mode, nine MUX1 will be selected instead of the multiplication operation. The positive and negative of the binary weight will determine whether the feature maps are reversed. After MUX2 and before DEMUX1, both modes will share the same computation units. The adder tree adds nine groups of 16-bit data each time and produces a 16-bit result for the convolution accumulation unit (CAU). The convolution results of each feature map channel will be accumulated on the CAU, and the final convolution result of the output feature map channel will be obtained after summing with bias in standard convolution mode or scaling with the scaling factor in the binary convolution mode. For ReLu module, it receives the result after the hybrid-precision CONV module and outputs the non-negative value after MUX4, which is judged by a comparator. For the pooling module, our DCNN engine currently only support average pooling. It caches and accumulates the results after the ReLu module with the buffer. When the arbiter judges that the configured pooling length is reached, the accumulated result goes through the shifter for a shifted division operation.

**Figure 8:**
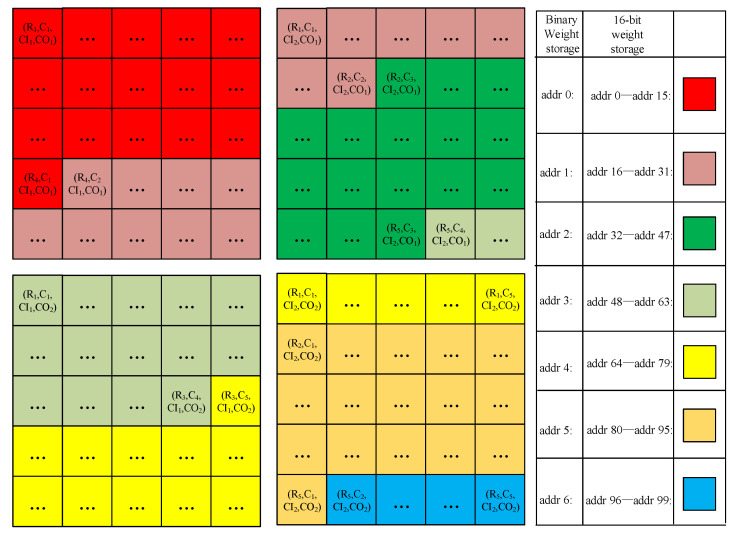
Hybrid-precision memory access.

In addition to a hybrid-precision convolution, we also implement a hybrid-precision memory access strategy. As shown in Figure 8, it can be seen how weights are stored in the hybrid-precision weight buffer. Taking weights of 5 × 5 × 2 × 2 size as an example, each small square represents each weight, and four large squares form a collection of all the weight data. R_i_, C_j_, CI_k_, and CO_t_ represent the weight data of row i, column j, input channel k, and output channel t, respectively. (R_i_, C_j_, CI_k_, CO_t_) represents the weight data of row i, column j, input channel k, and output channel t.

For demonstration, all the weight data can be divided into different color blocks, according to every 16 steps. As can be seen from the table of Figure 8, the area of each kind of color needs at most 16 addresses for weight data storage and 99 addresses for all the weight data storage while storing 16-bit weights. However, only one address is required for the area of each kind of color, and six addresses are required in total while storing binary weights. The advantage of this compact storage method is that larger neural networks and more weights can be stored in binary weight mode, compared with 16-bit weight mode. In addition, multiple binary weights can be read out in parallel, which reduces read time.

#### 3.2.2. Denoising Engine and SNR Sensing Engine

In the process of inference, the normalization and quantization are generally done off-chip. Here, we only discuss the implementation of denoising engine and signal sensing engine.

**Figure 9:**
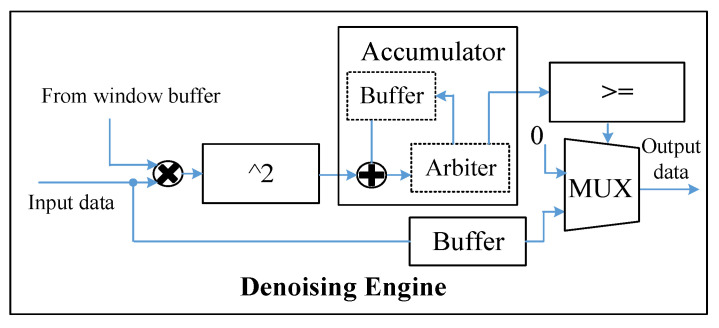
Denoising engine.

The structure of the denoising engine is shown in Figure 9. It consists of a multiplier, a squarer, an accumulator, a comparator, a multiplexer, and a buffer. The adder, buffer, and arbiter together form an accumulator. According to Equation (14) in the denoising algorithm, the input data are first multiplied with the window function in the multiplier. The square of its multiplication result is then accumulated in the accumulator. When the number of multiplication results is up to the window length, the short-time energy is obtained by the accumulator. In the comparator, the obtained short-time energy will be compared with a certain threshold. If the energy is above that certain threshold, the input data are considered as a valid signal, and the input data cached in the buffer is output via the multiplexer. Otherwise, the input data are considered as noise and the multiplexer selects zero data for output.

**Figure 10:**
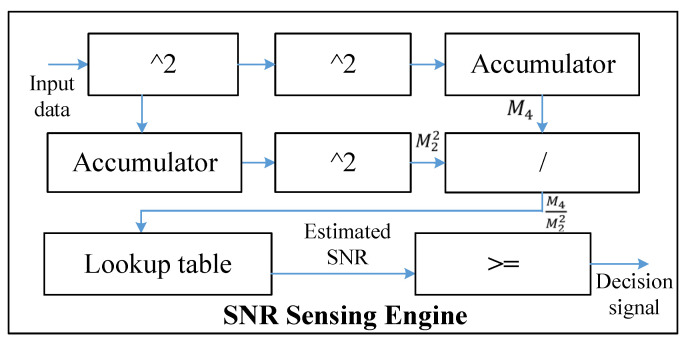
Signal sensing engine.

The structure of the signal sensing engine is shown in Figure 10. It consists of a divider, a lookup table, a comparator, two accumulators, and three squarers. According to Equation (1) in the SNR-aware precision reconfiguration algorithm, the SNR value will first be calculated by M2M4 estimation. It can be observed that the estimated SNR can be expressed as a function of an independent variable of $\left(\frac{M_{4}}{M_{2}^{2}}\right)$. This function can be implemented by a lookup table, which saves computational costs. Therefore, we only need to calculate $M_{2}^{2}$ and $M_{4}$ to obtain the final estimated SNR value. For the calculation of $M_{2}^{2}$, it is obtained by a squarer, an accumulator, and another squarer. For the calculation of $M_{4}$, it is obtained by one shared squarer, another squarer, and an accumulator. After computing $M_{2}^{2}$ and $M_{4}$, the estimated SNR can be inferred from the divider and the lookup table. In the comparator, the estimated SNR is compared with a certain threshold value, and a decision signal is generated. If the estimated SNR is above a certain threshold, the input data are considered as a high SNR signal, and the decision signal is pulled up. Otherwise, the input data are considered as a low SNR signal, and the decision signal is pulled down.

## 4. Experiments and Results

To validate the proposed DCNN processor, we have implemented it using a Zynq-7045 FPGA board.

### 4.1. Dataset

During our experiments, the publicly available UAV dataset [14] was chosen to validate our algorithm–hardware codesign performance, which is also convenient for comparison with existing work. Table 1 shows the composition of this dataset. In this dataset, a total of 227 segments of time-domain RF data were recorded, which can be classified into 10 types. One type is 10.25 s of background noise, and the other nine types are 5.25 s of RF data from three UAVs (AR, Bebop, and Phantom) in different flight modes, which include on and connected mode; hovering mode; flying mode; and flying with video recording mode. More details can be found in the article [29]. During training, we adopted the K-fold cross-validation method to randomly divide the dataset into 10 non-overlapping folds, of which 9 folds are used for training, and the remaining fold is used for testing. This process will be repeated 10 times by us, to evaluate the entire dataset.

### 4.2. SEI-DCNN Network Architecture

We use a 4-layer network architecture named SEI-DCNN for UAV identification. The detailed network structure of SEI-DCNN is shown in Table 2. Please note, we implemented the SEI-DCNN on our accelerator for demonstration, but different DCNNs can be implemented on our proposed DCNN engine by changing its user instructions.

### 4.3. Evaluation Method

We evaluate our proposed method from the three aspects, including accuracy, F1 score, and power efficiency.

- Accuracy and F1 score

In the classification task, there are generally four classification cases including true positive (*TP*), false positive (*FP*), true negative (*TN*), and false negative (*FN*) [30]. *TP* indicates the number of positive samples accurately predicted as positive. *FP* indicates the number of negative samples incorrectly predicted as positive. *TN* indicates the number of negative samples accurately predicted as negative. *FN* indicates the number of positive samples incorrectly predicted as negative. Based on the above, we use accuracy and F1 score to evaluate the performance of our SEI algorithm as follow:

(15) $$Accuracy=\frac{TP+TN}{TP+TN+FP+FN}$$

(16) $$F1\;score=2\times \frac{Precision\times Recall}{Precision+Recall}\;\;\;\;\;\;\;\;\;\;\;\;\;\;\;\;\{\begin{array}{c} Precision=\frac{TP}{TP+FP}\\ \;\;\;\;\;\;\;Recall=\frac{TP}{TP+FN} \end{array}$$

The accuracy focuses on the proportion of correct samples to all test samples, while the F1 score focuses on precision and recall.

- Power Efficiency

As one of the important and effective metric, power efficiency [31] is used to evaluate the performance of hardware, which considers not only speed but also power consumption. Specifically, GOPs/w is used to quantify power efficiency as follow:

(17) $$GOPs/w=\frac{GOPs}{P_{w}}$$

where $P_{w}$ indicates the power consumption that can be measured in watts by the power measurement tool of the software. *GOPs* can be used as a measure of speed, indicating the number of giga operations per second when the algorithm is running with specific hardware.

### 4.4. Test Setup

**Figure 11:**
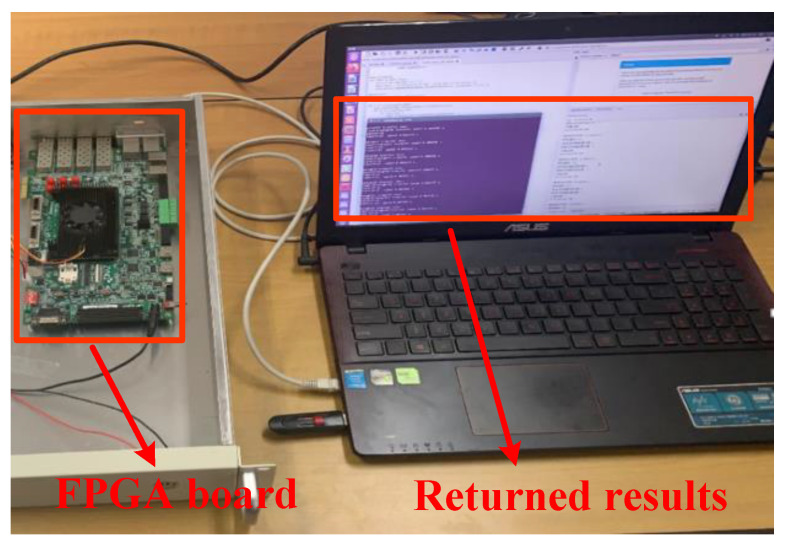
Test setup.

Figure 11 shows a test setup before the experiment. The bitstream of the hardware design is downloaded to the FPGA in advance via the serial port. The PC is responsible for configuring the SEI-DCNN on FPGA and sending the local offline RF samples to the FPGA. After the neural network completes the identification, the returned results are printed on the display screen of PC.

### 4.5. Experimental Results

**Figure 12:**
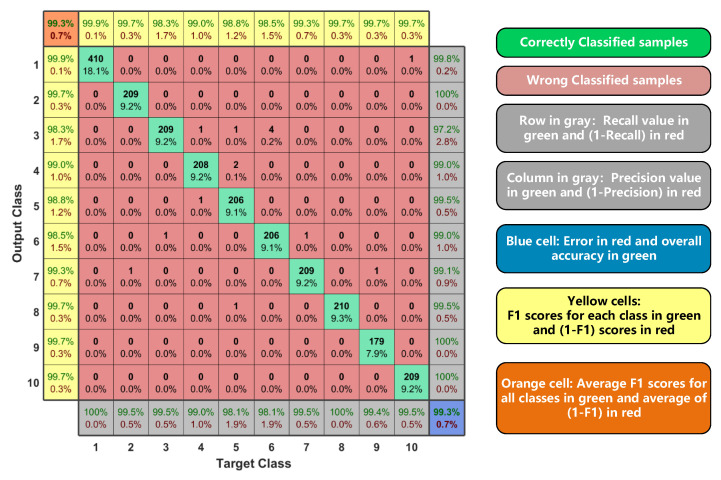
The confusion matrix of SEI-DCNN with FLOAT32 precision.

By counting the prediction results of DCNN for each category, we can obtain the confusion matrix of SEI-DCNN with FLOAT32 precision, as shown in Figure 12. The green cells and red cells represent the correctly and incorrectly classified samples, respectively. The yellow cells on the left and top side represent the F1 scores for each class. The gray cells on the right and bottom side represent the recall and precision value, respectively. According to the yellow blocks and gray blocks, we can obtain the average F1 scores in the orange cell and overall accuracy in the blue cell. Finally, both the classification accuracy and F1 score of our method are 99.3%.

**Figure 13:**
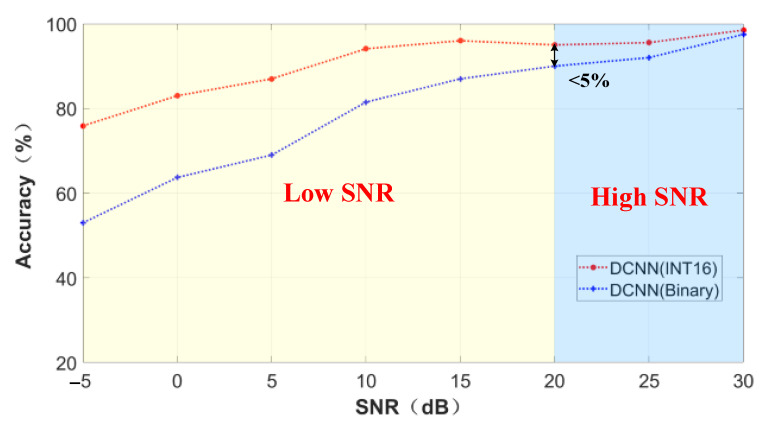
Classification accuracy under different SNR.

To evaluate the performance of our algorithm under different SNRs, we added noise to the original dataset to simulate the real situation. Figure 13 shows the classification accuracy under different SNR. We compare the accuracy of DCNN with INT16 precision and binary DCNN in the SNR range of [−5 dB, 30 dB]. The step size of the SNR interval is 5. It can be seen from the figure that the accuracy of DCNN is more than 5% higher than that of binary DCNN in the SNR range of [−5 dB, 20 dB). With the increase in SNR, the accuracy of binary DCNN in the SNR range of [20 dB, 30 dB] gradually approaches the accuracy of DCNN with INT16 precision. Especially, the accuracy of both neural networks can reach more than 97% at 30 dB, but binary DCNN has greater advantages in computing and storage. Based on the above analysis, we set 20 dB as our threshold in our SNR sensing engine to determine which DCNN is used.

Table 3 shows the detailed power consumption obtained from Vivado software, including static power and dynamic power. In the low SNR condition of [−5 dB, 15 dB], the power consumption of the FPGA is about 1280 mW with 16-bit weight mode. In the high SNR condition of [20 dB, 30 dB], the power consumption of the FPGA is about 610 mW with binary weight mode.

**Figure 14:**
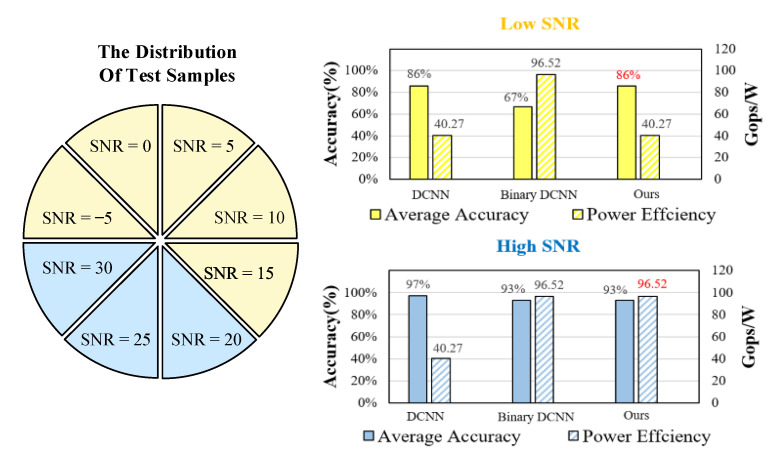
The histogram of average accuracy and power efficiency under the high SNR and low SNR.

By using 8 equally divided test samples under the low SNR range of [−5 dB, 15 dB] and high SNR range of [20 dB,30 dB], we can obtain the average accuracy and power efficiency of three methods, as shown in Figure 14. In the low SNR condition, the average accuracy of our method is 87%, which is 16% higher than that of binary DCNN. In the high SNR condition, our method has a higher power efficiency of 96.52 Gops/W compared with DCNN.

## 5. Comparison with Other Methods

For the algorithm, we compared our algorithm with four papers that use the same dataset in their implementations. As shown in Table 4, our algorithm can achieve an accuracy of 99.3% and a F1 score of 99.3% with FLOAT32 precision, which is better than those of other algorithms. Besides, our algorithm can achieve an accuracy of 98.5% and an accuracy of 97.5% with INT16 precision and binary precision, respectively, which is still high among these algorithms.

For hardware, we compared our accelerator with four designs including CPU, GPU, and FPGAs, as shown in Table 5. Compared with CPU- and FPGA-based designs, our design has higher computational performance. Although our computational performance is not as good as the GPU due to fewer computing resources, our design achieved the highest power efficiency of 40.12 Gops/W and 96.52 Gops/W with INT16 precision and binary precision, respectively.

## 6. Conclusions

In this work, we have proposed a SEI hardware accelerator with a SAS-SEINet algorithm co-designed for UAV surveillance, which has been implemented on a Zynq-7045 FPGA board. In terms of the algorithm, we propose a SAS-SEINet including signal preprocessing, a SNR-aware precision reconfiguration, and a scalable SEI neural network. In terms of hardware, a SNR sensing engine, denoising engine, and specialized DCNN engine with hybrid-precision convolution and memory access are designed for SAS-SEINet acceleration. The final results show that the accuracy of 99.3% and the F1 score of 99.3% are the best among the state-of-the-art algorithms. The power efficiency of 40.12 Gops/W and 96.52 Gops/W can be achieved with INT16 precision and binary precision, respectively, which are the highest compared with the other hardware designs.

## Figures and Tables

**Table 1:** Composition of UAV dataset.

UAV	Label	Type-10	Segments	Samples	Ratio
Bebop	2	On and connected	21	420 × 10^6^	9.25%
	3	Hovering	21	420 × 10^6^	9.25%
	4	Flying	21	420 × 10^6^	9.25%
	5	Flying with video recording	21	420 × 10^6^	9.25%
AR	6	On and connected	21	420 × 10^6^	9.25%
	7	Hovering	21	420 × 10^6^	9.25%
	8	Flying	21	420 × 10^6^	9.25%
	9	Flying with video recording	18	360 × 10^6^	7.93%
Phantom	10	On and connected	21	420 × 10^6^	9.25%
No UAV	1	Background noise	41	820 × 10^6^	18.06%

**Table 2:** SEI-DCNN network architecture.

Layer	Embedded Structure	Output Shape	Parameter
0	Input Layer	(None, 16,384, 1)	-
1	STFT-Conv	(None, 128, 128, 1)	65,280
2	Conv2D + average pooling	(None, 64, 64, 32)	320
3	Conv2D + average pooling	(None, 16, 16, 64)	18,496
4	Flatten + FC	(None, 10)	163,850

**Table 3:** The power consumption on FPGA.

SNR (dB)	Weight Precision	Static Power	Dynamic Power	Total Power
[−5, 15]	INT16	245 mW	1035 mW	1280 mW
[20, 30]	Binary	245 mW	365 mW	610 mW

**Table 4:** The comparison of our algorithm with existing algorithms.

Method	Accuracy	F1 Score
[14]	46.8%	43.0%
[15]	59.2%	55.1%
[13]	95.4%	95.0%
[17]	98.4%	98.3%
[12]	99.2%	99.1%
Ours	99.3% ^1^	99.3% ^1^
	98.5% ^2^	98.4% ^2^
	97.5% ^3^	97.3% ^3^

^1^ is obtained with FLOAT32 precision, ^2^ is obtained with INT16 precision, and ^3^ is obtained with binary precision.

**Table 5:** The comparison of our hardware design with other designs.

Design	Platform	Weight Precision	Complexity (Mop)	Time (µs)	Computational Performance (Gops)	Chip Power (W)	Power Efficiency (Gops/W)
CPU	INTEL I5-6500	FP32	64.23	4610.91	13.93	30.29	0.46
GPU	NVIDIA GTX 1660	FP32	64.23	301.21	213.24	14.02	15.21
MILCOM2019 [19] (FPGA)	XCZU9EG	INT16	0.36	24.00	15.18	1.15	13.17
ISCAS2021 [32] (FPGA)	ZCU104	INT16	0.89	26.78	33.08	0.85	38.92
Ours (FPGA)	XC7Z045	INT16 Binary	64.23 64.23	1246.12 1090.77	51.54 58.88	1.28 0.61	40.27 96.52

## Data Availability

The data used in this study are available online at https://data.mendeley.com/datasets/f4c2b4n755/1 (accessed on 1 November 2020).

## Abbreviations

The following abbreviations are used in this manuscript:

UAV	Unmanned Aerial Vehicle
RF	Radio Frequency
SEI	Specific Emitter Identification
ML	Machine Learning
DCNN	Deep Convolution Neural Network
DNN	Deep Neural Network
UHF	Ultra-High Frequency
USRP	Universal Software Radio Peripheral
STFT	Short-time Fourier Transform
SNR	Signal-to-Noise Ratio
KLD	Kullback-Leibler Divergence
M2M4	Second and Fourth Moments
FPGA	Field Programmable Gate Array
CPU	Central Processing Unit
GPU	Graphic Processing Unit
